# 

**Published:** 1909-07

**Authors:** 


					﻿Sixty=fourth Year. j
- V0L.XXIV.	JULY, 1909.	No. 12
BUFFALO
MEDICAL JOURNAL
Established 1845 by AUSTIN FLINT M. D\
---------!---- \ }
V s G o i
EDITOR:
WILLIAM WARREN POTTER, M. D.
ASSISTANT EDITORS:
WILLIAM C. KRAUSS, M. D.	NELSON W. WILSON, M. D
ASSOCIATE EDITORS:
JAMES WRIGHT PUTNAM, M. D. ERNEST WENDE, M.D.
JOHN PARMENTER, M. D.	JOHN A. MILLER, Ph. D.
MAUD J. FRYE, M. D.
				

